# ­Using the *C. elegans lem-2* Gene to Reconstruct the Human LEMD2 Mutation Associated with Hutterite-type Cataract/Cardiomyopathy

**DOI:** 10.17912/micropub.biology.000273

**Published:** 2020-06-29

**Authors:** Ayaa AlKhaleefa, Frances L. Snider, Henry J. Duff, James D. McGhee

**Affiliations:** 1 1. Department of Biochemistry and Molecular Biology, Alberta Children’s Hospital Research Institute, Cumming School of Medicine, University of Calgary, Calgary, Alberta CANADA; 2 Cardiac Sciences, Libin Cardiology Institute, Cumming School of Medicine, University of Calgary, Calgary, Alberta CANADA

**Figure 1:**
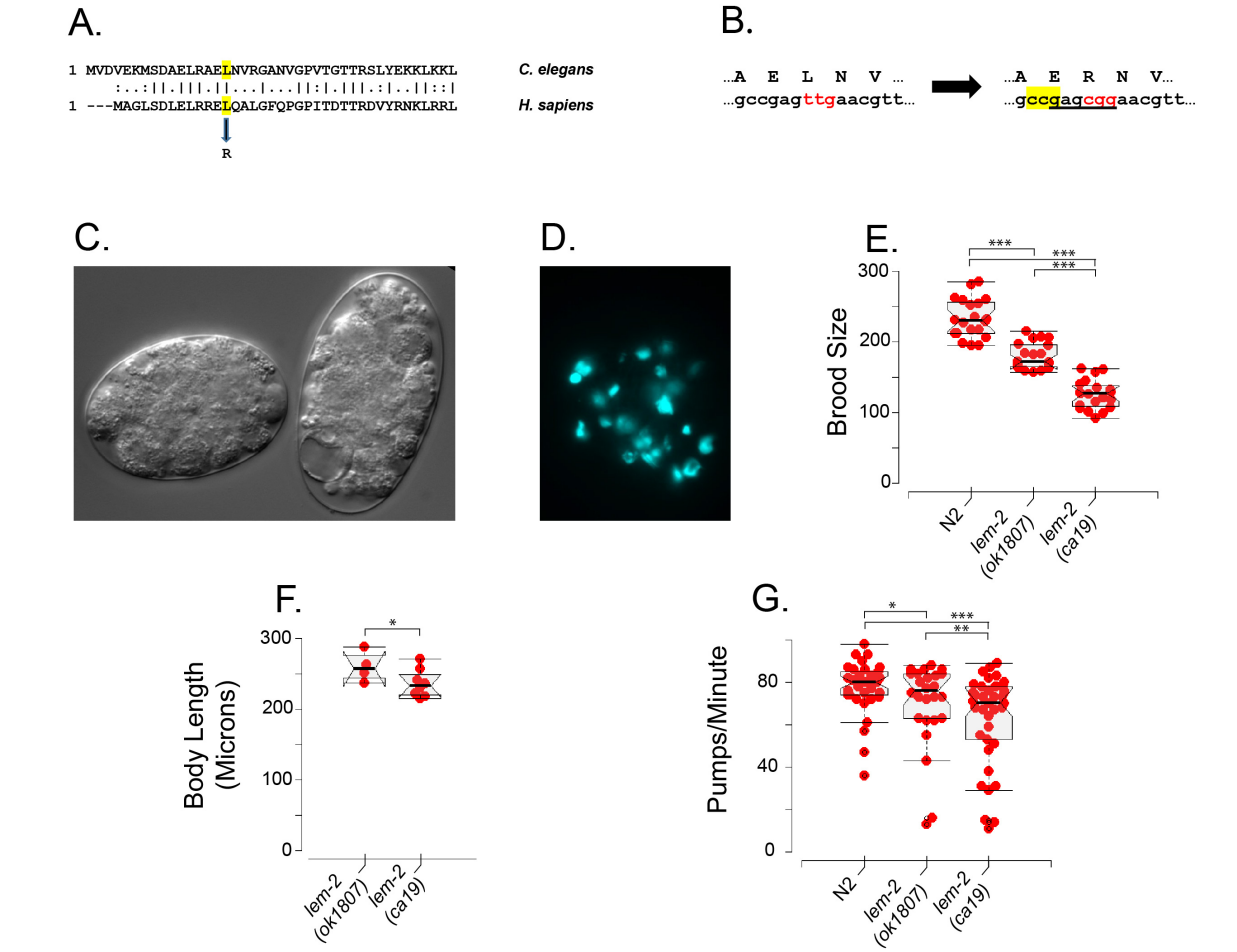
**A.** Needleman-Wunsch alignment of the N-terminal region of the Human LEMD2 protein and the *C. elegans* LEM-2 protein. Highlighted in yellow is the conserved Leucine residue that is changed to Arginine in the human mutation. **B.** Schematic summary of the CRISPR-induced changes in the *C. elegans* *lem-2* gene introducing the Leucine to Arginine mutation at amino acid residue 16. Protein sequence is shown above the DNA sequence. The leucine ttg codon is changed to arginine cgg codon (red), thereby introducing a *BsrbI* restriction site (underlined). The reverse complement of the CRISPR/Cas9 PAM sequence is highlighted in yellow. **C.** Differential interference contrast (DIC) image of typical arrested *lem-2(ca19)* embryos whose mothers had been treated with *emr-1* RNAi. **D.** DAPI-stained JM311 *lem-2(ca19)* embryo whose mother had been treated with *emr-1* RNAi. **E.** Total brood sizes measured for 4-5 days following the L4 stage for strains N2 (wildtype); VC1317 *lem-2(ok1807)* and JM311 *lem-2(ca19)*. “***” indicates t-test probability <<0.001. Beeswarm-boxplots were assembled in RStudio. **F.** Body length measurements (microns) measured for arrested non-Green L2 larvae segregating from strains JM312 and JM313, i.e. *emr-1(gk119); lem-2(ok1807) or emr-1(gk119); lem-2(ca19)* larvae segregating from mothers homozygous for the respective *lem-2* allele but heterozygous for *emr-1(gk119)*. “*” indicates the (one-sided) t-test probability that *lem-2(ca19)* larvae are shorter than *lem-2(ok1807)* < 0.05. Beeswarm-boxplots were assembled in RStudio. **G.** Rates of pharyngeal pumping measured in strains N2 (wildtype), VC1317 and JM311. Beeswarm-boxplots were assembled in RStudio. The three lowest pumping rates for each data set were assessed by RStudio boxplot as “outliers” and were omitted from significance tests. T-test probabilities are indicated as follows: “*” < 0.05; “**” < 0.01; “***” < 0.0001.

## Description

The human LEMD2 protein and its homologs in other animals are associated with the inner nuclear membrane, the nuclear lamina and with functions such as chromatin organization and nuclear repair (Barton *et al.* 2015). The human mutation (c.38T>G; L13R) changes a single amino acid in the highly conserved LEM domain and, when homozygous, is associated with juvenile cataracts and with a greatly increased incidence of early onset cardiac arrest (Shokeir and Lowry 1985; Boone *et al.* 2016; Abdelfatah *et al.* 2019). The carrier frequency of this mutation in the North American Hutterite population is estimated to be as high as 12% (Abdelfatah *et al.* 2019).

The LEMD2 homolog in *C. elegans* is LEM-2. The *lem-2* gene has been well characterized and appears to be largely redundant with the gene *emr-1* (Lee *et al.* 2000; Liu *et al.* 2003; Barkan *et al.* 2012; Cohen-Fix and Askjaer 2017). Although *lem-2* knockouts, whether by gene deletion or by administration of RNAi, show only mild phenotypes, ablation of both *lem-2* and *emr-1* genes causes complete lethality: if both zygotic and maternal contributions are removed, animals arrest as early embryos; maternally rescued animals arrest at ~ the L2 larval stage (Lee *et al.* 2000; Liu *et al.* 2003; Barkan *et al.* 2012; Cohen-Fix and Askjaer 2017). We have reconstructed the “Hutterite-type cataract/cardiomyopathy” mutation in the *C. elegans* *lem-2* gene and now compare mutant phenotypes to the phenotypes produced by complete *lem-2* knockouts. A longer term aim will be to exploit this reconstructed mutation in *C. elegans* to identify LEM-2 interacting factors, both biochemically and genetically.

A partial sequence alignment (Figure 1A) shows that amino acid leucine 16 in the *C. elegans* LEM-2 protein corresponds to amino acid leucine 13 in human LEMD2. As shown in Figure 1B, we used CRISPR-Cas9 methods (Dokshin *et al.* 2018) to convert *C. elegans* leucine 16 to Arginine 16 (codon change from TTG to CGG); the mutation, designated *lem-2(ca19*), introduced a *BsrbI* restriction site that was used to follow the mutant gene through genetic crosses, including two initial outcrosses. The *lem-2(ca19)* mutation acts similarly to a complete *lem-2* gene knockout: treating the otherwise quite healthy strain JM311 *lem-2(ca19)* with *emr-1* RNAi by feeding (Kamath and Ahringer 2003; Kamath *et al.* 2003) leads to 100 % lethality, as previously reported for the *lem-2* deletion allele *tm1582* (Barkan *et al.* 2012) and that we now also confirm for the *lem-2* deletion allele *ok1807* used as our positive control. The key phenotype (Figure 1C) is embryonic arrest with fewer than 100 cells, frequent vacuoles and no obvious sign of differentiation (standard gut granule birefringence assay); a typical arrested embryo stained with DAPI (1 µg/ml) shows irregular condensed nuclei and occasional anaphase bridges (Figure 1D), much as has been previously reported for the complete *lem-2(tm1582)* knockout (Barkan *et al.* 2012) and as we now also confirm for the *lem-2(ok1807)* deletion. Administering *emr-1* RNAi by injection (generally recognized as more effective than feeding) into JM311 quickly leads to maternal sterility, consistent with LEM-2/EMR-1 requirements in the germline. At this point, we conclude that the *lem-2(ca19)* mutation behaves as if it approximates a null.

We now looked more closely at the phenotypes of the *lem-2* mutations in the presence of wildtype *emr-1* function. Figure 1E shows that both *lem-2* alleles *ok1807* and *ca19* show decreased brood size compared to N2 wildtype animals. However, the *ca19* brood size is significantly lower than is the brood size of the complete *lem-2* knockout, i.e., the *ca19* phenotype is “worse” than the null phenotype. Thus *ca19* could be described as a mild antimorph (Muller 1932) and this antimorph classification will be explained and defended below. A mild antimorphic nature of allele *ca19* is also revealed by measurements of the pure zygotic effect of *lem-2* loss. Following the scheme described in (Barkan *et al.* 2012), we produced a balanced strain in which *lem-2(ca19)* was homozygous and the *emr-1(gk119)* deletion was heterozygous, balanced with the chromosomal translocation *hT2green* (see **Methods** for full genotype)*.* 1/16 of the offspring of this balanced strain are homozygous for both *lem-2(ca19)* and *emr-1(gk119)* and arrest as non-Green L2 larvae. As shown in Figure 1F, the nose-to-tail length of arrested *emr-1(gk119); lem-1(ca19)* larvae) produced by *emr-1(+)* mothers is detectably lower than for the control *emr-1(gk110); lem-2(ok1807)* complete knockout larvae, again consistent with *lem-2(ca19)* behaving as a mild antimorph. Figure 1G shows that both *lem-2* mutations show a slightly lower pharyngeal pumping rate; once again, the *ca19* phenotype is slightly more severe than that of the complete gene knockout. The slower pumping rates shown by both mutants agree with previous observations made with the *lem-2(tm1582)* allele (Barkan *et al.* 2012).

In summary, we have produced a mutation in the *C. elegans* *lem-2* gene reconstructing a LEMD2 mutation that causes juvenile cataracts and premature cardiac arrest in the North American Hutterite population. Our main conclusion is that, in *C. elegans*, this single amino acid mutation acts similarly to a complete loss of function mutation; however, it also appears to show a mild antimorphic character. LEM-domain proteins are known to be multifunctional, binding to other proteins (e.g. BAF and lamins), the nuclear membrane and even DNA (Barton *et al.* 2015). Thus, if the *ca19* mutation compromises one LEM-2 function but not others, the mutant protein could form non-productive complexes that could interfere with wildtype function or with the redundant function of EMR-1, in other words act as an antimorph; we have not tried to test this model by assessing phenotypes of heterozygotes. In any case, even if this mutation can act like an antimorph in *C. elegans,* there is no guarantee that the corresponding mutation acts as an antimorph in humans; for example, molecular interactions with other components of the inner nuclear membrane could be different in humans and in worms.

## Methods

Strain JM311 *lem-2(ca19*) was produced as described above (Figure 1B and (Dokshin *et al.* 2018)), including two outcrosses to N2 wildtype worms and validation by sequencing of PCR amplified fragments. Strain VC1317 *lem-2(ok1807)* was obtained from the *Caenorhabditis* Genetics Centre and outcrossed once (the strain designation was not changed). To assess the phenotype of maternally rescued *emr-1; lem-2* larvae, we constructed strains in which the *lem-2* allele was homozygous but the *emr-1* deletion allele was heterozygous and balanced by a reciprocal translocation for which we use the shorthand *hT2green.* The proper designation of *hT2green* is *hT2[bli-4(e937) let-?(q782) qIs48] (I;III)* where the integrated chromosomal insertion *qIs48* *[Pmyo-2::gfp; Ppes-10::gfp; Pges-1::gfp]* results in GFP expression. The relevant strains used in this experiment are as follows: JM312 *emr-1(gk119)/hT2green I; lem-2(ok1807) II; +/hT2green III.* JM313 *emr-1(gk119)/hT2green I; lem-2(ca19) II; +/hT2green III.* To measure the tip-to-tail length of arrested larvae, small non-Green animals (picked at a time when the rescued Green animals on the plate were young adults) were suspended in egg buffer containing 0.2% Tricaine + 0.02% Tetramisole + 5mM Sodium Azide and mounted on agarose pads; DIC images were analyzed using ImageJ. Pumping rates were measured at room temperature with one day adults in the presence of 10mM serotonin in 10% M9 buffer mixed with an equal volume of overnight culture of *E. coli* OP50 (Weeks *et al.* 2018). Pumping rates were measured by video recording at a magnification of 20X and analyzed at slower frame rates. RNAi by feeding was performed as described by (Kamath and Ahringer 2003), using library clone M01D7.6. Double stranded RNA was made by *in vitro* transcription of the same plasmid and injected at a concentration of 1 mg/ml as previously described (Fukushige *et al.* 2005; Goszczynski and McGhee 2005).

## Reagents

Strains JM311, JM312 and JM313 will be made available at the *Caenorhabditis* Genetics Center.
